# A Fast and Automatic Method for Leaf Vein Network Extraction and Vein Density Measurement Based on Object-Oriented Classification

**DOI:** 10.3389/fpls.2020.00499

**Published:** 2020-05-05

**Authors:** Jiyou Zhu, Jiangming Yao, Qiang Yu, Weijun He, Chengyang Xu, Guoming Qin, Qiuyu Zhu, Dayong Fan, Hua Zhu

**Affiliations:** ^1^Key Laboratory for Silviculture and Conservation of Ministry of Education, Key Laboratory for Silviculture and Forest Ecosystem of State Forestry Administration, Research Center for Urban Forestry, Beijing Forestry University, Beijing, China; ^2^Forestry College, Guangxi University, Nanning, China; ^3^Research Institute of Tropical Forestry, Chinese Academy of Forestry, Guangzhou, China; ^4^Inspection Department of Guangxi Medical College, Nanning, China

**Keywords:** leaf vein network, leaf vein density, object-oriented method, remote sensing, extraction

## Abstract

Rapidly determining leaf vein network patterns and vein densities is biologically important and technically challenging. Current methods, however, are limited to vein contour extraction. Further image processing is difficult, and some leaf vein traits of interest therefore cannot be quantified. In this study, we proposed a novel method for the fast and accurate determination of leaf vein network patterns and vein density. Nine tree species with different leaf characteristics and vein types were applied to verify this method. To overcome the image processing difficulties at the microscopic scale, we adopted the remote object-oriented classification method applied comprehensively in the field of remote sensing research. The key to this approach is to determine the universally applicable leaf vein extraction threshold values (scale parameter, shape parameter, compactness parameter, brightness feature, spectral feature and geometric feature). Based on our analysis, the following recommended threshold values were determined: the scale parameter was 250, the shape parameter was 0.7, the compactness parameter was 0.3, the brightness feature value was 230∼280, the spectral feature value was 180∼230, and the geometric feature value was less than 2. With the optimal extraction parameters applied, the extraction precision was above 96.40% on average for the nine species studied. The leaf vein density calculation rate increased by more than 87.3% compared to that of the traditional methods. The results showed that this method is accurate, fast, flexible and complementary to existing technologies. It is an effective tool for the fast extraction of vein networks and the exploration of leaf vein characteristics, particularly for large-scale studies in plant vein physiology.

## Introduction

The leaf vein network, an important morphological structure, is widely distributed on the leaf surface. It affects the mechanical support, water balance and resource circulation of the entire leaf (Wright et al., 2004; Brodribb and Holbrook, 2005; Lavorel and Grigulis, 2015; Cai et al., 2018). One important trait of the network is vein density, the total length of the leaf vein per unit area, which is a crucial index among the functional traits of the vein networks (Nardini et al., 2003). The leaf vein network characterizes the distribution, arrangement and orientation of the veins in the leaves, which is closely related to the transport efficiency of water and photosynthetic products during transpiration and photosynthesis (Nardini et al., 2003; Fu and Chi, 2006; Walls, 2011; Zhang et al., 2018). In addition, leaves are the most important functional organs of plants for photosynthetic carbon capture, and the morphological and functional diversification of plant leaves are often reflected by the high diversity of vein networks (McKown and Dengler, 2009; Feldman et al., 2017).

In many instances, leaf vein density has important theoretical significance for understanding leaf formation and adaptation mechanisms. It also provides a mechanism to explain the general trend of global plant ecological geography (Blonder et al., 2010; Brodribb and Field, 2010; Sack and Scoffoni, 2013). The research on the network structure of veins can be traced to 1861. Ettingshausen, an international ecologist, applied the vein network structure to plant classification (Hickey, 1973; McKown et al., 2010). For a long time, the leaf vein network structure has played an important role in plant classification. Different plant groups have diverse types of vein network structures (Hickey, 1973; Melville, 1976; Sieburth, 1999). For example, dichotomous venation is more common in pteridophytes, and the leaf vein system of dicotyledons is mostly netted venation, while parallel venation appears only in monocotyledons (Sack and Frole, 2006). In recent years, studies on the functional traits of leaf veins and their relationships with leaf water, leaf photosynthetic capacity and leaf carbon construction have received extensive attention from international researchers (Beerling and Franks, 2010; Blonder et al., 2010; Roth-Nebelsick et al., 2001; Sack et al., 2012). With the deepening of research, a pattern is emerging between the functional traits of the vein network and the environment, as well as the relationship between the functional traits of the vein network and the evolutionary trends of the plant system (Boyce et al., 2009; Field et al., 2011; Sack and Scoffoni, 2013). In addition, the vein network is important for understanding the mechanism of plant regulation of the environment (Field et al., 2011). Studies have shown that when plants are susceptible to adverse environmental stress, they often adapt to environmental changes by adjusting their functional traits (Corpataux et al., 2002; Kang et al., 2007; Mcelwain et al., 2016). For example, in a high-temperature environment or one in which water resources are very scarce, plants usually have a well-developed vein network structure, ensuring water supply balance and improving plant survival adaptation and competitiveness (McKown and Dengler, 2009; Baylis et al., 2013). In-depth study of leaf vein functional traits and their ecological characteristics is of far-reaching significance for exploring plant responses to global climate change. Furthermore, the field applications of research on leaf vein network functional traits are expanding. Plant functional traits are of great significance for community species composition, species ecological responses, maintenance of plant diversity, and material cycling in ecosystems (Lavorel and Garnier, 2002; Hooper et al., 2005; Price et al., 2014). Therefore, in-depth study of the functional properties of the vein network and its ecological characteristics is of great significance for predicting the response of plants and ecosystems to global changes (McGill et al., 2006; Violle et al., 2007; Webb et al., 2010). Based on previous studies, we found that veins contain important physiological information for plants. Quantification of the vein network is the primary prerequisite to the study of leaf vein traits, as well as one of the key steps in plant modeling and plant identification. Therefore, it is particularly important to study an efficient and fast method to quantify vein networks.

With the development and popularization of computer image processing technology, quickly extracting the required information from an image has become a popular research topic in the field of remote sensing (Sezgin and Sankur, 2004; He et al., 2009; Blaschke, 2010; McKown et al., 2010). Based on previous research, we found that image analysis software such as Photoshop and Image J were generally used in the calculation of leaf vein structure (Hüve et al., 2010; Kengni et al., 2016). These methods were not only time-consuming and laborious but also likely to cause great human error. At present, the research objects for leaf vein quantification are mainly digitized scans of fresh leaves, and the methods are mostly based on artificial neural networks, directional energy and K-means clustering (Nardini and Jansen, 2013; Price et al., 2014; Bühler et al., 2015). Some research is limited to the extraction of vein contours, and the measurement of its related indicators (e.g. leaf vein density) cannot be achieved (Zhang et al., 2015). Obtaining the leaf vein density still needs to be done with the traditional method, which increases the workload unnecessarily. Compared with using a flatbed digital scanner (Price et al., 2014), leaf vein images taken by an optical compound microscope provides richer information, such as shape, spectrum and brightness. eCognition is the world’s first object-oriented classification software (Vallis and Colton, 1996). The object-oriented method can fully consider the features of the target object, including brightness features, spectral features, geometric features and texture features. After multiscale segmentation of the image, a series of subunits that do not cross each other and do not overlap each other is formed, which reduces the fragmentation rate of the object (Fayad et al., 2002; Jing and Cheng, 2012). Therefore, in this study, we proposed a novel method of leaf vein extraction. We regarded vein microscopic images as remote-sensing images and explored the application of remote-sensing image processing technology in the classification and extraction of veins to calculated leaf vein density.

In this study, vein images of nine common deciduous and evergreen species representing both monocotyledons and dicotyledons (*Populus tomentosa* Carr., *Sophora japonica* L., *Fraxinus pennsylvanica* Var., *Koelreuteria paniculata* Laxm., *Acer truncatum* Bunge, *Ailanthus altissima* (Mill.) Swingle, *Caryota ochlandra* Hance, *Cordyline fruticosa* (L.) A. Cheval, and *Rhapis excelsa* (Thunb.) Henry ex Rehd.) with different leaf areas, leaf growth characteristics, vein types, covering monocotyledons and dicotyledons were taken as research objects. Large-scale vein images were identified, classified and extracted based on brightness features, spectral features and geometric features to achieve efficient quantification of leaf vein density.

## Training Sample Acquisition and Extraction Process

### Leaf Image Acquisition

The sampling sites in this study are located at Beijing Forestry University and the Guangxi University campus. Beijing, the capital of China, is located between longitudes 115°125’ and 117°130’ E and between latitudes 39°28’ and 41°05’ N. The climate is typical of the semi-humid continental monsoon climate in the northern temperate zone. The annual average temperature is 10∼14°C, the average summer temperature is 27.5°C, and the annual average precipitation is approximately 600 mm. Nanning City, the capital of Guangxi Province, China, is located between longitudes 107°45’ and 108°51’E and between latitudes 22°13’ and 23°32’N. The climate belongs to a subtropical humid monsoon climate. The annual average temperature is 12.8∼28.2°C. The average annual precipitation is 1300 mm. As shown in Table 1, to eliminate the difference in leaf vein extraction caused by leaf growth characteristics, nine tree species with different leaf sizes, leaf textures, leaf vein definitions and vein types in typical tree species used for urban greening with new growth were selected as training samples. The healthy mature leaves of the canopy were randomly collected in August 2018 in fine weather from 09:00 to 11:00. A total of 30 trees of each species were selected, and 10 leaves per tree were gathered from all cardinal directions. We used a traditional method for acquiring a leaf image (soak + microscope photography method). The leaf samples were cleared of chlorophyll by soaking in a 5% NaOH solution for 7 days in a dark environment until the leaf was transparent (the solution was changed every 24 h). We added 1 drop of toluidine blue (TB) stain to make a temporary slide after rinsing it with distilled water. We placed the slides under an optical microscope (Leica DM6000B, Monroe, LA, United States) at 40 × magnification (2.2013 × 1.6468 mm. In this experiment, three pictures of each slide were randomly taken for experimental analysis. A total of 120 images were collected from each tree species.

**TABLE 1:** Leaf traits and leaf vein characteristics of nine tree species with different leaf areas, leaf thicknesses, leaf textures, pubescence, leaf vein clarity (visibility of vein on leaf surface) and vein types.

**Tree species**	**Leaf area /cm^2^**	**Leaf thickness/mm**	**Leaf texture**	**Pubescence**	**Vein clarity**	**Vein type**
*Populus tomentosa* Carr.	(101.38 ± 19.32)^b^	(0.23 ± 0.12)^b^	Thick oriaceous leaf	Smooth leaves	Clear	Netted venation
*Acer truncatum* Bunge	(30.50 ± 4.21)^d^	(0.15 ± 0.04)^c^	Oriaceous leaf	Smooth leaves	Clear	Netted venation
*Fraxinus pennsylvanica* Var.	(21.82 ± 3.24)^*e*^	(0.21 ± 0.08)^b^	Paper-based leaf	Trichome-covered leaves	Clear	Netted venation
*Ailanthus altissima* (Mill.) Swingle	(36.99 ± 6.14)^d^	(0.24 ± 0.06)^b^	Thin oriaceous leaf	Trichome-covered leaves	Clear	Netted venation
*Koelreuteria paniculata* Laxm.	(23.24 ± 2.35)^*e*^	(0.18 ± 0.06)^c^	Paper-based leaf	Trichome-covered leaves	Blurry	Netted venation
*Sophora japonica* L.	(8.93 ± 1.50)^f^	(0.16 ± 0.03)^c^	Paper-based leaf	Trichome-covered leaves	Blurry	Netted venation
*Caryota ochlandra* Hance	(98.23 ± 15.30)^b^	(0.26 ± 0.05)^b^	Thin oriaceous leaf	Smooth leaves	Blurry	Parallel venation
*Cordyline fruticose* (L.) A. Cheval	(128.46 ± 21.53)^a^	(0.33 ± 0.13)^a^	Grass-based leaf	Trichome-covered leaves	Blurry	Parallel venation
*Rhapis excelsa* (Thunb.) Henry ex Rehd.	(58.66 ± 11.24)^c^	(0.25 ± 0.10)^b^	Thin oriaceous leaf	Smooth leaves	Blurry	Parallel venation

*Different lowercase letters in the table mean significant differences at *p* < 0.05 level.*

### Vein Image Processing

#### Extraction Process

**FIGURE 1:**
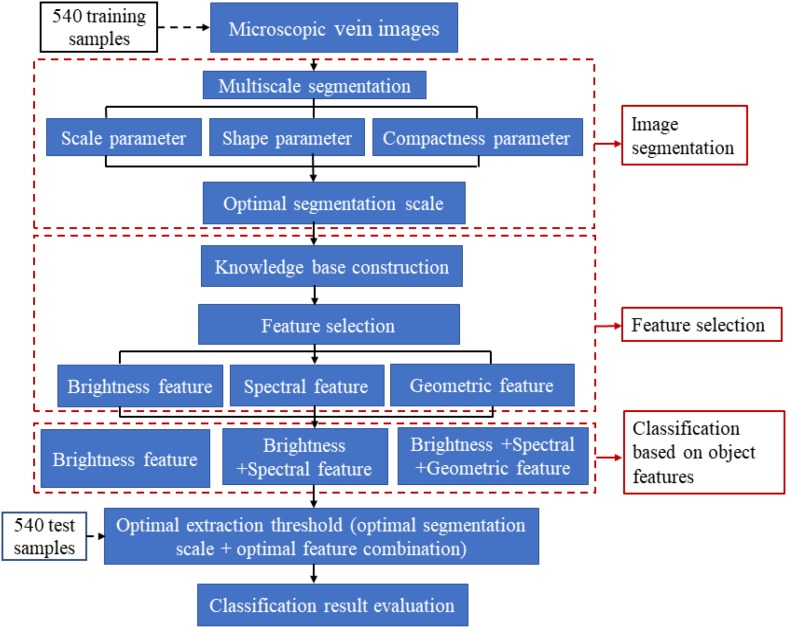
Process of leaf vein extraction and optimization based on object-oriented classification. This operation is generally divided into three main steps: image segmentation, feature selection and classification based on object features.

The remote-sensing classification method is performed with eCognition Developer 64 software. It takes a pixel object containing multiple spatial relationships among semantic information as the processing unit. This tool can classify higher-level remote-sensing images and extract target object information (Schönmeyer et al., 2006). Therefore, we can use such relationship features in the software to make a distinction between the veins and background in the image. For instance, veins have a special strip shape as well as unique spectral information compared with the background in microscopic images. As shown in Figure 1, the object-oriented taxonomy consists of two main functional modules, the image segmentation module and the target object extraction module. The extraction process of the vein network mainly includes steps such as image preprocessing, multiscale segmentation, knowledge base construction, feature selection and leaf vein extraction (Schnack et al., 2001; Schönmeyer et al., 2006). The purpose of image segmentation is to generate target objects, which is the most critical step in object-oriented classification (Schnack et al., 2001). Segmentation methods based on object-oriented methods mainly include chessboard segmentation, quadtree segmentation, contrast segmentation, multiscale segmentation, spectral difference segmentation, multi-threshold segmentation and contrast filter segmentation (Biberthaler et al., 2003). Multi-scale segmentation uses the region-growing segmentation algorithm. It can divide the image into several subregions that do not intersect each other. This is to have the same characteristics according to the heterogeneity of the target object and non-target object (Mckinney and Cai, 2002). Multiscale segmentation is the most commonly used segmentation method in eCognition’s object-oriented classification technology and plays a critical role in image segmentation (Feng, 2010). According to the leaf vein characteristics of the tree species, the reason we choose the multiscale segmentation method in this study is to achieve the maximum weight homogeneity of the target object after segmentation. In the multiscale segmentation process, the segmentation parameters mainly include scale parameters, shape parameters and compactness parameters. To ensure the best fit of the vein objects, we need to constantly adjust the scale, shape and compactness parameters, i.e. the segmentation threshold.

### Object-Oriented Leaf Extraction and Optimization

#### Image Preprocessing

To enhance the contrast between the veins and the background of the microscopic image, we performed a 1% linear stretch of the visual parameters on all training samples in the eCognition software to ensure the relative consistency of the initial features of all training samples to achieve better segmentation.

#### Image Segmentation

**FIGURE 2:**
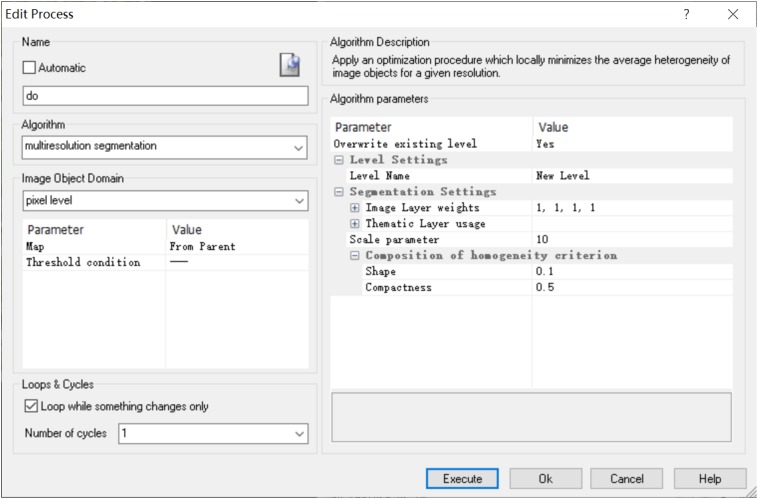
Leaf vein multiscale segmentation interface based on eCognition software.

Image segmentation is the most critical step in object-oriented taxonomy. The quality of segmentation in this process directly influences the accuracy of extraction (Nguyen et al., 1999; Feng, 2010). Because the characteristics of the leaf veins are different from those of the background, we choose the multiscale segmentation method here. It is the most effective method of information acquisition at different scales. This uses the region-merger method with the least heterogeneity of the object and the non-object to minimize the weight heterogeneity of the segmented veins. The object-oriented method can separate regions of arbitrary resolution according to the characteristics of different objects, thus extracting objects of different scales (Jing and Cheng, 2012). Therefore, we need to constantly adjust the segmentation parameters to control the threshold of the merging algorithm to find the optimal image segmentation parameters (Figure 2).

#### Leaf Vein Extraction Optimization

**FIGURE 3:**
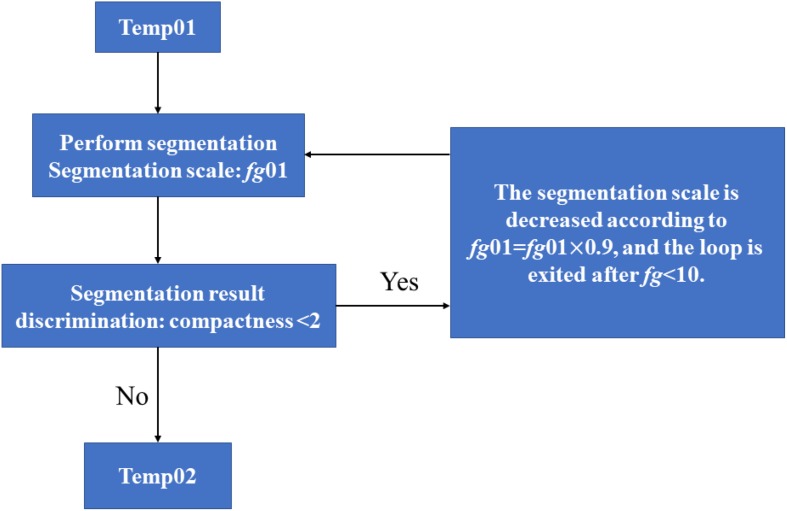
Flow of the generation of a straight leaf segment in the eCognition software. “Temp01” is the name of the initial vein object set in the software, and “Temp02” is the final spliced complete vein.

As shown in Figure 3, except for *Populus tomentosa* Carr., *Acer truncatum* Bunge, *Caryota ochlandra* Hance and *Rhapis excelsa* (Thunb.) Henry ex Rehd., which were glabrous, all the species tested were pubescent. Therefore, during the process of photographing the vein microscopic image, the veins may be discontinuous or interrupted due to the occlusion from leaf hairs. To reduce the error caused by such phenomena, we need to splice the interrupted vein parts into a complete network. In this paper, we used the “circulating growth method” to determine the direction of interrupted veins. This method was used by the object-oriented classification technology of the eCognition software to find a universal rule and threshold range for leaf vein extraction.

##### Generating a straight vein segment

The leaf vein objects called temp01 were divided into several straight leaf veins. Only in this way could we ensure the correct direction of vein growth. As shown in Figure 3, the steps were as follows: (1) Set the initial segment scale parameter *fg*01 = 150. (2) Set the segmentation algorithm as multiscale segmentation. The segmentation parameters were as follows: the segmentation scale value of *fg*01, the shape parameter of 0.7 and the compactness parameter of 0.9. The purpose was to generate a leaf object that was as straight as possible. (3) Among the generated temp01 class objects, the objects with a compactness parameter <2 were classified as temp02 and no longer participated in the classification. (4) The division scale was set to self-decreasing, and then *fg*01 = *fg*01 × 0.9 was set. (5) The loop was set. When *fg*01 > 10, the program would return to step (2) to continue segmenting; otherwise, it would exit the loop. By choosing this segmentation algorithm, the leaf object (temp01) could be broken down to generate a straight temp02 class. The segmentation process is reproduced below (Figure 3).

##### Leaf vein growth

**FIGURE 4:**
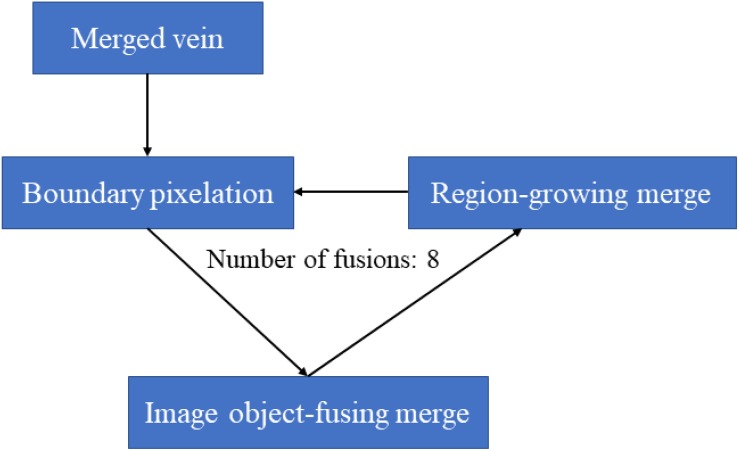
Flow of the growth of interrupted veins in eCognition software. This fusion growth is to connect the segmented leaf veins.

The background objects around the veins were of different shapes and sizes, making it difficult to carry out fusion growth. Here, the purpose of fusion was to connect interrupted veins. We used pixelated boundary objects to enable the veins to grow correctly in this study. The growth process is shown in Figure 4.

The specific steps were: (1) Combine the background classes into one object. (2) At the boundary between the background object and the temp02 class, to make the boundary pixelized, we used chessboard segmentation to divide the background object into one group of pixels, which was convenient for leaf vein growth. (3) We used the image object fusion algorithm to grow the interrupted vein. Then, we set the length fitting function as follows: the target weight was 1, the seed weight was −1, the candidate’s background weight was 0, and the length fitting function threshold was Length > 0 Pxl. The length fitting function was TL-SL > 0 (TL was the target object length, and SL was the seed object length). The software automatically performed calculations based on this function to obtain the best blending method so that the straight objects in the temp02 class would grow in the main direction. (4) The region-growing algorithm was set to integrate the temp02 class into the background object class, which was adjacent to the temp02 class, and merge it into temp02. The purpose of this step was to speed up the integration process, in particular to integrate vein objects that were removed as speckles. The background objects were repeatedly divided and merged by the above rules, and the process was stopped after eight cycles.

### Calculation of Leaf Vein Density and Accuracy Analysis

The traditional measurement method of leaf vein density is based on image-processing software such as Photoshop and Image J. The biggest defect of these methods is that they need to calculate vein one by one, which not only takes a lot of time, but also brings large human error, especially for images with higher vein density. In this study, we adopt the remote-sensing object-oriented method approach to calculate leaf vein density. After determining the optimal extraction threshold for the veins in the eCognition software, we could measure the leaf vein density in batches by selecting the category of classified objects under the related class (length-classified objects).

A total of 120 microscopic leaf images were randomly selected from each type of tree for analysis. A total of 60 images per tree species were used for object-based classification for batch calculation, and the remaining 60 images were measured using traditional measurement methods (Image J software). We compared our technique of vein extraction to one that uses Image J. The Image J extraction technique involves tracing veins manually using the polygon selection tool. Using the root-mean-square error, the accuracy of the results was tested (Feng, 2010) according to

(1) $$R=\sqrt{\frac{1}{n}\,\sum_{i=1}^{n}\left(P_{1}-P_{0}\right)}$$

(2) $$P^{\prime }=\frac{{\bar{P}}_{1}-R}{\bar{P_{1}}}\times 100\%$$

In the formulas, the following abbreviations are used: n—Number of samples, *P*′—Extraction accuracy, *P*_1_—Measured value (traditional method), ${\bar{P}}_{1}-$Measured mean (traditional method), *P*_0_—Extracted value, *R*—Root-mean-square error.

## Results and Analysis

### Scale Parameters

**FIGURE 5:**
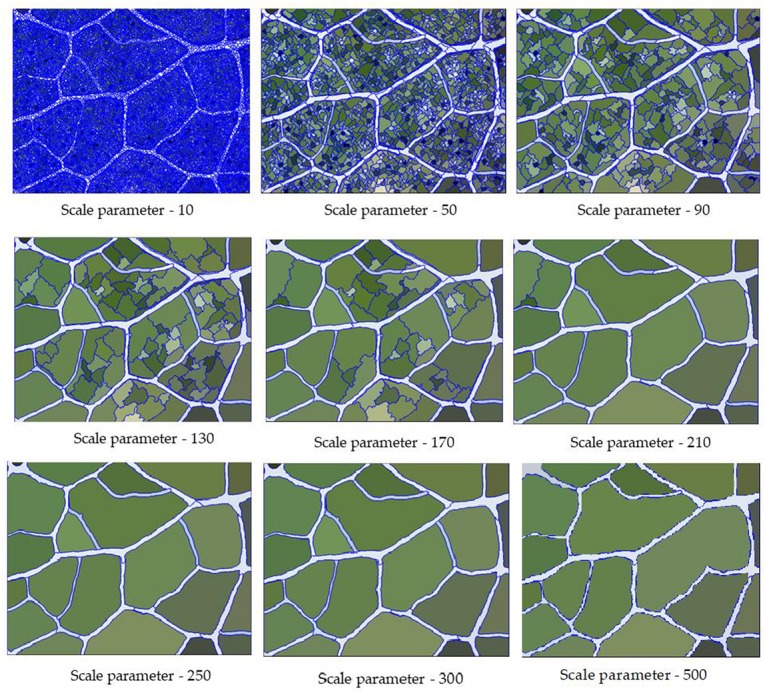
Segmentation results of veins under different scale parameters. Setting the scale parameter too small or too large will affect the segmentation accuracy of vein.

The scale parameter is the most critical segmentation parameter of multiscale segmentation, and it directly affects the segmentation of the entire image. Table 2 shows that there were significant differences in the accuracy of the final extraction of leaf veins by different scale parameters (*P* ≤ 0.05), but there were no significant differences among different tree species (*P* > 0.05). As shown in Figure 5, each parameter in the image segmentation affects the segmentation result to a certain extent. Therefore, according to the characteristics of each class of objects, the segmentation parameters should be reasonably selected and set to make the segmentation effect more noticeable. When the scale parameter was set to be too small, the internal image was broken to a large extent. It not only prolonged the segmentation process but also caused the characteristic difference in the target object (the leaf vein) to be strongly influenced by the non-target object, thereby reducing the degree of separation between categories. Conversely, when the scale parameter was set to be too large, the image segmentation was relatively rough, and the boundary of the veins was low. At the same time, under large-scale conditions, non-leaf vein structures around the veins were merged into the target object, which greatly reduced the accuracy of the segmentation and the final classification accuracy.

**TABLE 2:** Difference analysis of vein image segmentation results under different segmentation parameters and extraction rules.

**Source of variation**	**Inter-parameters**		**Inter-species**	
	***F***	***P***	***F***	***P***
Segment parameters	43.102	0.0001	23.134	0.8680
Brightness features	68.322	0.0005	29.302	0.3851
Spectral features	35.021	0.0029	26.001	0.3871
Geometric features	20.105	0.0001	13.134	0.8402

**F* is the calculated test statistic used to assess differences between groups. *P-*value is an indicator to measure if there is a difference between the control group and the experimental group.*

In this study, we set scale parameters of 10, 50, 90, 130, 170, 210, 250, 300, and 500 for the segmentation test. Through the comparison of the measured results, the classification accuracy of the veins was found not to be linear with the size of the segmentation scale. After continuous adjustment, the classification accuracy was the highest when the scale was 250.

### Shape Parameters

**FIGURE 6:**
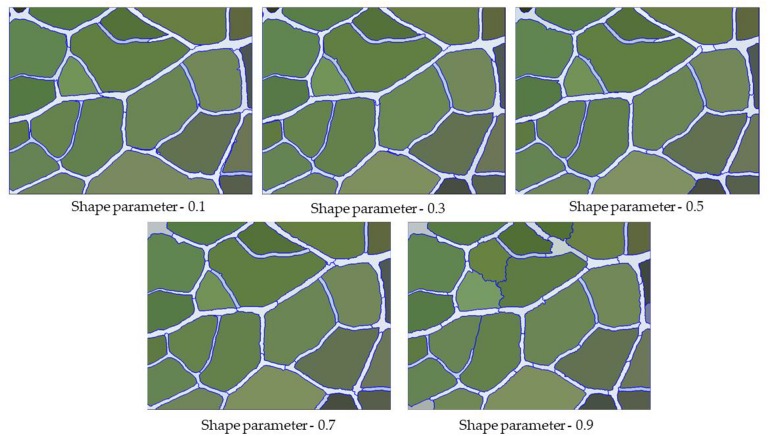
Segmentation result of veins under different shape parameters.

The shape parameter is the weight that the shape standard should have when defining the segmentation image, which determines the boundary fit for the image object. In general, the higher the value, the smaller the effect of color during the segmentation process. After the first step of multiscale segmentation and the determination of the optimal scale parameters, we adjusted the shape parameters with the optimal scale parameter of 250 and then determined the optimal shape parameter threshold. In the range of 0∼1, we found that the degree of coincidence of the leaf vein boundary first increased and then decreased with increasing shape parameters. Under the condition that the scale parameters were certain, we set the shape parameter to 0.1, 0.3, 0.5, 0.7, and 0.9. As can be observed in Figure 6, when the shape parameter weight was set to be too small, the entire image boundary was very fragmented, and many background objects were segmented. Conversely, when the shape parameter weight was set to be too large, this reduced the spectral homogeneity inside the vein, causing some of the veins to be incorrectly grouped with the background object. After the repeated adjustment of parameters, we found that when the shape parameter was 0.7, the segmentation effect on the vein contour was best (Figure 6).

### Compactness Parameters

The compactness parameter is used to define the weight of the compactness standard, which determines the degree of compactness in the objects generated after image segmentation. In general, the higher the value is, the more compact the boundaries of the image object. In the range of 0∼1, the segmentation coincidence degree of the vein network first increases and then decreases with the increase of the compactness parameter. Therefore, after determining the optimal scale parameters and shape parameters, we continuously adjusted the compactness parameters to maximize the segmentation quality.

**FIGURE 7:**
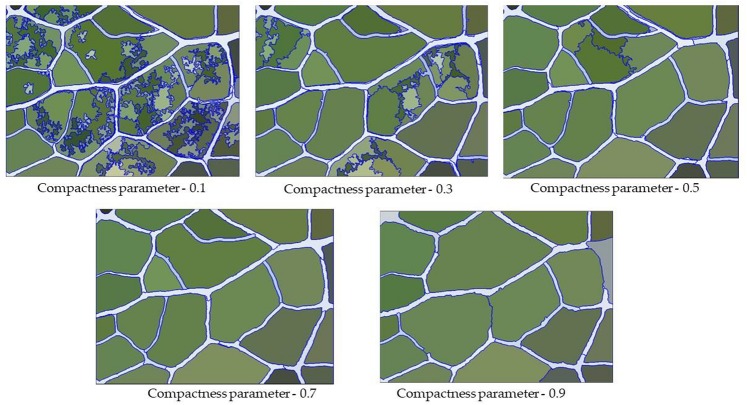
Segmentation result of veins under different compactness parameters.

**FIGURE 8:**
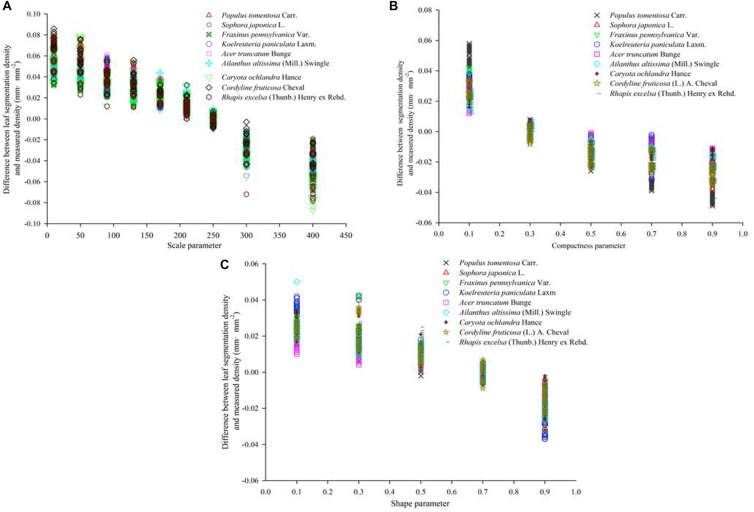
Segmentation accuracy of nine tree species at different segmentation scales. The value of ordinate refer to the difference between the density calculated by eCognition software and the density calculated by Image J software. **(A–C)** show the vein density under different scale parameters, different shape parameters and different compactness parameters, respectively.

The compactness parameters define the weight of the compactness criterion. The higher the value is, the more compact the image objects may be. As shown in Figure 7, when the compactness parameter weight was set to be too small, although the boundary coincidence degree was relatively high, some veins were not divided. In contrast, when the compactness parameter weight was set to be too large, this led to a dense partitioning interface as well as many mis-segmentations. Therefore, we needed to constantly adjust the parameters and observe the quality of segmentation under different parameters. We found that when the compactness parameter was 0.3, the segmentation quality of the vein network was highest (Figure 8).

### Knowledge Base Construction, Leaf Extraction and Optimization

In the vein extraction process, knowledge base construction is essential for the extraction of the image after segmentation. The vein micrograph contains a variety of information that can be used to classify the target object (Table 3). After generalizing and describing the characteristics of the veins, we converted these characteristics into extraction rules to perform the extraction of veins. The main classification features of microscopic image objects included brightness features, spectral features, geometric features and texture features. Based on the characteristics of the veins and microscopic images of leaves of different morphologies, textures and types, we selected brightness features, spectral features and shape features to construct a vein extraction knowledge base. These three features were transformed into the rules for the extraction below.

**TABLE 3:** Image object information on brightness, spectral and geometric features.

**Characteristic category**	**Main content**	**Main application**
Brightness features	Contrast, Saturation, Lightness, Shade, Tone balance	Target object recognition of the most basic features
Spectral features	NDVI(Normalized Difference Vegetation Index), NDWI (Normalized Difference Water Index), Mean, Standard deviation, Ratio, HIS(Hue, Intensity and Saturtaion)	The most important features of target object recognition and extraction
Geometric features	Length, Width, Area, Length/Width, Border length	Mainly reflects the geometry of the target object

#### Brightness Features

**FIGURE 9:**
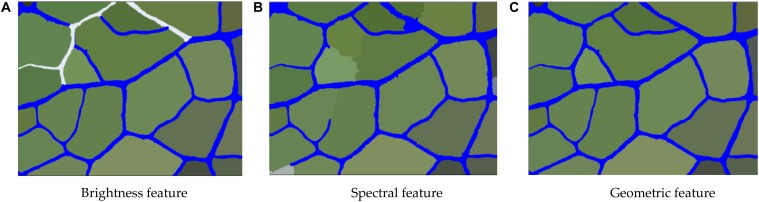
The extraction results of the vein network based on brightness characteristics, spectral features and geometric features. **(A)** Brightness feature **(B)** Spectral feature **(C)** Geometric feature.

In the eCognition software, all the objects in the vein image were divided into two main types–the leaf vein and background. The selected leaf vein unit showed the brightness value of the vein. After summarizing the luminance of all the vein units, the maximum and minimum values were used as the extraction threshold range. Table 2 shows that the threshold value of the brightness rule has a significant difference in the extraction precision of the veins, and there was no significant difference among different tree species. As shown in Figure 9A, after repeated adjustment of 540 images, it was found that the brightness of the veins could largely distinguish the target object (vein) from the background when the brightness of the veins was in the range of 230 to 280. In addition to the veins, we found that there were more lustrous mesophyll backgrounds that were also divided, most of which belonged to dense stomata or guard cell contour boundaries. For leathery leaves, the veins were prominent, but the developed stomatal structure also formed a line that was relatively consistent with the veins on the microscopic image. At this point, there were more false positives, and it was difficult to extract the vein objects by brightness features only. The reason for this may be that there were some differences in the brightness of the microscopic images due to the uneven thickness of the leaves. Therefore, we needed to further extract the vein network by means of extra features.

#### Spectral Features

Table 2 shows that the threshold value of the spectral feature rule has a significant effect on the extraction precision of the veins (*P* ≤ 0.05), but there was no significant difference among different environments and different tree species (*P* > 0.05). As shown in Figure 9B, the process of vein extraction still shows partial misclassification or leakage based on the brightness features, so it was necessary to further classify the veins according to the spectral features. Through the analysis of the microscopic image properties, we found that the red band threshold of the vein was 180∼230, while the red spectral band value of the background was largely below 185. Therefore, by setting the spectrum (red band value) to 180 or more, the veins could be separated from the background to a large extent. However, when the veins were extracted based on spectral features, we found that a very small number of veins were still not extracted or were mistaken for the background. We suspect that this phenomenon may be due to other tissue structures around the veins, such as trichomes, stomata and other impurities, overlapping with the spectral characteristics of the veins. Therefore, to eliminate such phenomena, we need to further extract the veins using geometric features.

#### Geometric Features

Table 2 shows that the threshold value of the geometric feature rule has a significant effect on the extraction precision of the vein (*P* ≤ 0.05), but there was no significant difference among different environments and different tree species (*P* > 0.05). Since it was difficult to completely separate the veins from the background by relying solely on the brightness and spectral features, geometric features had to be used for further extraction. Unlike the shape contours of structures such as stomata in the background, the veins have a rather long linear contour. Therefore, the special shape features of the veins in the image could be used to further extract the vein network. First, all the vein objects were merged in the category of brightness feature extraction. Then, the merged leaf object features were more obvious; the separated objects were linear, while the background objects were mostly rectangular. The density was further extracted with these shape characteristics, and the closer the vein shape was to linearity, the smaller the density value was. As shown in Figure 9C, the leaf vein images of the nine species were repeatedly tested, the category with a geometric feature value <2 was classified as the vein so that most of the background objects could be removed, and most of the merged veins were completely retained.

### Leaf Vein Extraction Optimization

**FIGURE 10:**
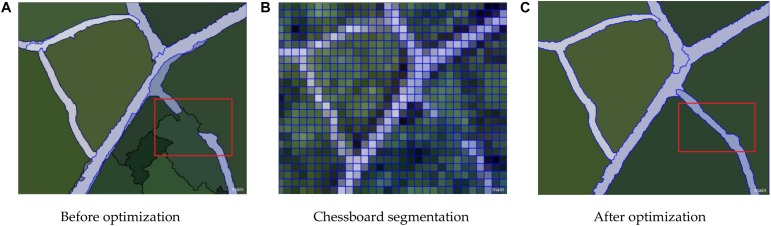
Leaf vein network optimization results. **(A)** Before optimization **(B)** Chessboard segmentation **(C)** After optimization.

**FIGURE 11:**
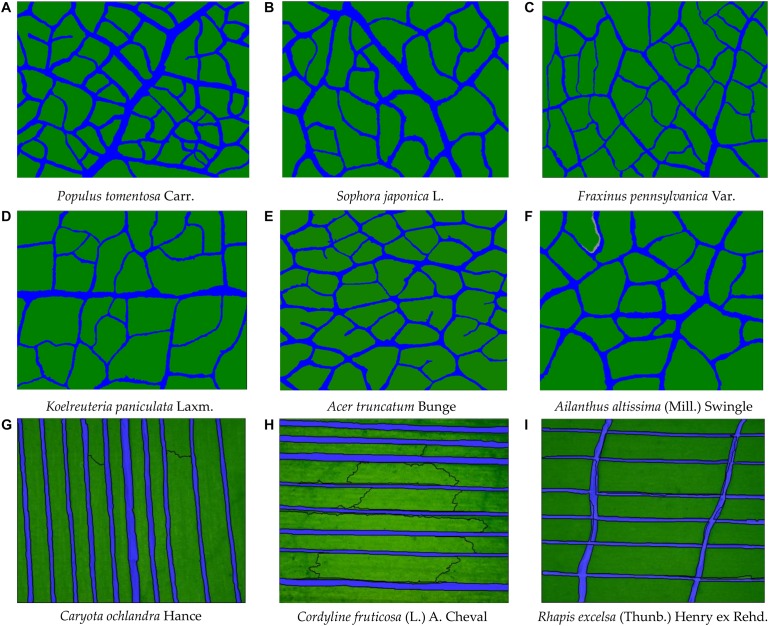
Extraction results of leaf vein images of nine tree species at the optimal extraction threshold. The blue part is the vein, and the green part is the mesophyll. All colors are set in the software. **(A)** Populus tomentosa Carr. **(B)** Sophora japonica L. **(C)** Fraxinus pennsylvanica Var. **(D)** Koelreuteria paniculata Laxm. **(E)** Acer truncatum Bunge **(F)** Ailanthus altissima (Mill.) Swingle. **(G)** Caryota ochlandra Hance **(H)** Cordyline fruticosa (L.) A. Cheval **(I)** Rhapis excelsa (Thunb.) Henry ex Rehd.

The result is presented in Figures 10, 11. After different segmentation parameters, layer superposition and optimization splicing of extraction rules, the extraction accuracy was gradually improved according to the optimized identification of the nine tree species.

### Extraction Accuracy Analysis

**FIGURE 12:**
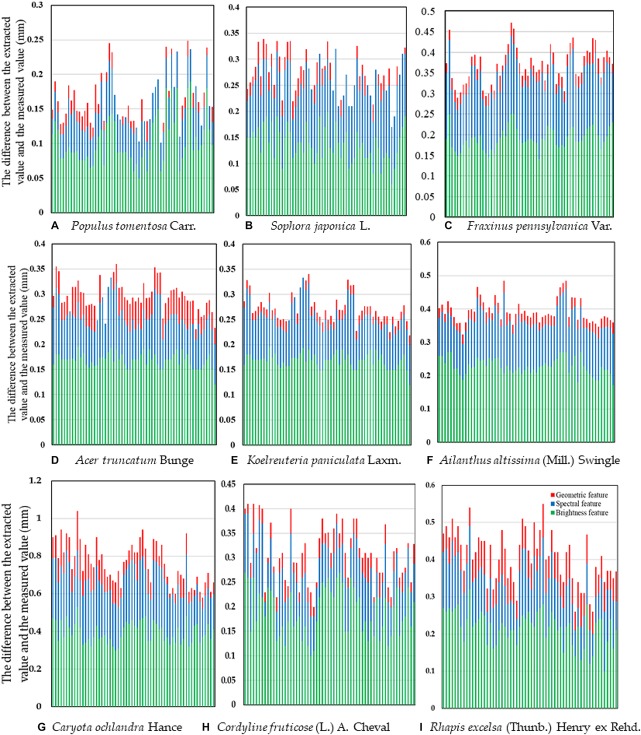
Vein extraction accuracy based on multiple features. The *X*-axis is a single test image sample. **(A)** *Populus tomentosa* Carr. **(B)** *Sophora japonica* L. **(C)** *Fraxinus pennsylvanica* Var. **(D)** *Acer truncatum* Bunge **(E)** *Koelreuteria paniculata* Laxm. **(F)** *Ailanthus altissima* (Mill.) Swingle **(G)** *Caryota ochlandra* Hance **(H)** *Cordyline fruticose* (L.) A. Cheval **(I)** *Rhapis excelsa* (Thunb.) Henry ex Rehd.

To obtain universally applicable extraction parameters, we selected nine common green plant leaves with different leaf sizes, leaf textures, leaf surface features and vein types as training samples and thoroughly applied large-scale techniques to solve microscale problems. At the same time, we fully considered the unique growth characteristics of leaves, such as the problem of leaf vein discontinuity caused by blade pubescence, and used cycle iteration to splice the intermittent veins to ensure the integrity of the vein network. A random selection of sixty images in each type of tree were measured one by one using the traditional Image J method, and the results of the automatic extraction of the object-oriented classification method were compared and verified. As shown in Figure 12 and Table 4, although the leaf characteristics and leaf vein density of the nine tree species were significantly different, the final extraction results were not affected, and the extraction accuracy, on average, was above 96.0%. At the same time, there was no difference among the nine tree species we studied. This showed that through the proper adjustment of the extraction parameters, the object-oriented method could be used to extract the vein network and calculate the leaf vein density. In addition, we found that the rate of calculating leaf vein traits using object-oriented classification techniques was much better than that of traditional measurement methods (Image J software). As shown in Table 5, the extraction rates of nine different tree species, for one picture, all increased by more than 87.3%, and the highest increase was 94.9%. The results showed that this method is accurate, fast, flexible and complementary to existing technologies. It is an effective tool for the fast extraction of the vein network.

**TABLE 4:** Analysis of the extraction value of the leaf vein.

**Tree species**	**Extraction value (mm⋅mm^–2^)**	**Measured value (mm⋅mm^–2^)**	**Difference value (mm⋅mm^–2^)**	**Accuracy (%)**
*Populus tomentosa* Carr.	0.6936 ± 0.4110	0.6794 ± 0.1302	0.0142 ± 0.0034	97.95
*Acer truncatum* Bunge	0.8686 ± 0.3241	0.8373 ± 0.3621	0.0313 ± 0.0026	96.40
*Fraxinus pennsylvanica* Var.	1.1272 ± 0.3569	1.1006 ± 0.4853	0.0266 ± 0.0032	97.64
*Ailanthus altissima* (Mill.) Swingle	0.8933 ± 0.1753	0.9104 ± 0.2423	0.0171 ± 0.0013	98.12
*Koelreuteria paniculata* Laxm.	1.1972 ± 0.3934	1.1631 ± 0.5164	0.0541 ± 0.0036	97.15
*Sophora japonica* L.	1.2251 ± 0.2543	1.2542 ± 0.5521	0.0291 ± 0.0040	97.68
*Caryota ochlandra* Hance	2.3294 ± 0.6731	2.2378 ± 0.7224	0.0916 ± 0.0045	96.07
*Cordyline fruticose* (L.) A. Cheval	1.3467 ± 0.4357	1.3213 ± 0.6734	0.0254 ± 0.0012	98.11
*Rhapis excelsa* (Thunb.) Henry ex Rehd.	1.9659 ± 0.5804	1.8956 ± 0.7866	0.0703 ± 0.0025	96.42

*The extraction value is the leaf vein density that is automatically extracted based on object-oriented classification, and the measured value a is the leaf vein density calculated by the traditional measurement method (Image J software).*

**TABLE 5:** Comparison of the calculation rate of the traditional measurement method (Image J software) and the new method (eCognition software) for one picture.

**Tree species**	**Leaf vein measurement time**		**Increased rate /%**
	**Traditional method /min**	**New method /s**	
*Populus tomentosa* Carr.	2.37	8	94.9
*Acer truncatum* Bunge	2.32	8	94.7
*Fraxinus pennsylvanica* Var.	2.46	8	95.2
*Ailanthus altissima* (Mill.) Swingle	2.15	8	94.1
*Koelreuteria paniculata* Laxm.	2.56	8	95.5
*Sophora japonica* L.	1.95	8	94.8
*Caryota ochlandra* Hance	1.21	8	90.1
*Cordyline fruticose* (L.) A. Cheval	1.16	8	89.5
*Rhapis excelsa* (Thunb.) Henry ex Rehd.	1.03	8	87.3

*The increased rate is the percentage increase in the calculation rate of the new method over that of the traditional measurement method.*

## Conclusion

In this study, we proposed a novel method for the fast and accurate determination of leaf vein network patterns and vein density. To overcome the image processing difficulties at the microscopic scale, we adopted the remote object-oriented classification method applied comprehensively in the field of remote-sensing research. A total of nine tree species with different leaf characteristics and vein types were tested to verify this method. The key to this approach is to determine the leaf vein extraction threshold values (scale, shape, compactness, brightness, spectral characteristic, and geometric feature) that are universally applicable. Based on our analysis, the following recommended threshold values were determined: the scale parameter was 250, the shape parameter was 0.7, the compactness parameter was 0.3, the brightness feature value was 230∼280, the spectral feature value was 180∼230, and the geometric feature value was less than 2. We found that although the leaf growth characteristics and vein traits are quite different, the threshold values we obtained for the tree vein network extraction and leaf vein density calculation of these species achieved a high precision. The extraction precision was above 96.40% on average for the nine tree species studied. The calculation rate of leaf vein traits increased by more than 87.3% compared to that of the traditional measuring methods. The proposed method is an effective tool for the fast extraction of vein networks and the exploration of leaf vein characteristics, particularly for large-scale studies in plant vein physiology.

## Data Availability Statement

Some datasets generated for this study are available on request to the corresponding author. All models used during the study appear in the submitted article. The copyright of the computing software (eCognition) used in this article should be purchased from the official website.

## Author Contributions

JZ conceived and designed the study and performed the experiments. JZ, HZ, JY, QY, QZ, and CX contributed to materials and tools. JZ, GQ, CX, QY, DF, and WH contributed to the data analysis and manuscript preparation. JZ and DF contributed to the modification of the manuscript. All authors read and approved it for publication.

## Conflict of Interest

The authors declare that the research was conducted in the absence of any commercial or financial relationships that could be construed as a potential conflict of interest.
